# Reviewed article: Rossignoli P, Pontarolo R, Fernandez-Llimos F. Variability in public procurement prices for Group 1A drugs in the specialized pharmaceutical component: an observational study, 2013-2022. Epidemiol Serv Saude. 2025:34;e20240733

**DOI:** 10.1590/S2237-96222025v34e20240733.a

**Published:** 2025-09-01

**Authors:** William Neves Oliveira

**Affiliations:** 1 Universidade Federal de São João del-Rei, São João del-Rei, MG, Brazil Universidade Federal de São João del-Rei São João del-Rei MG Brazil

## Peer review A

## Questionnaire

Does the manuscript contain new and significant information to justify publication? Yes

Does the Abstract (Summary) clearly and accurately describe the content of the article? Yes

Is the problem significant and concisely stated? Yes 

Are the methods described comprehensively? Yes

Are the interpretations and conclusions justified by the results? Yes

Is adequate reference made to other work in the field? Yes

## Manuscript Structure

Length of article is: Adequate

Number of tables is: Adequate

Number of figures is: Adequate

**Please state any conflict(s) of interest that you have in relation to the review of this paper (state “none” if this is not applicable).**

None

**Interest:** Good

**Quality**: Excellent

**Originality:** Average

**Overall:** Good

**Recommendation:** Minor Revision

## Comments

Gostaria de elogiar os autores pelo esforço em conduzir um estudo sobre um tema importante. O manuscrito contribui para a compreensão de evidências do mundo real sobre as diferenças nos preços de aquisição de medicamentos do componente especializado obtidos pelo Ministério da Saúde versus pela Secretaria Estadual de Saúde do Paraná. Apesar de observado pouquíssimas sugestões de melhorias e esclarecimentos adicionais, espero ajudar a melhorar a qualidade e a clareza do estudo, garantindo o seu máximo impacto.

Título

A palavra “diferenças” no título, poderia ser substituída por “variabilidade”. Sugiro tal alteração para um aspecto de cunho mais científico.

Introdução

A introdução está bem escrita, fundamentada e apresenta coesão lógica entre os parágrafos.

Métodos

I. No trecho “Apesar de ser um estudo observacional, não foi utilizada a iniciativa Strengthening the reporting of observational studies in epidemiology para estruturar os métodos porque muitos dos tópicos previstos não seriam aplicados neste estudo, nomeadamente, participantes, viés, tamanho do estudo”, é necessário ao menos colocar o nome da iniciativa em itálico e inserir sua sigla (STROBE).

II. A seção de análise de dados é bem escrita, no entanto, gostaria que as fórmulas referentes a cada cálculo fossem apresentadas. A visualização apenas em texto é até compreensível, mas exige do leitor diversas releituras, o que pode ser mitigado pela união de texto + fórmulas.

III. Por que os preços de aquisição não foram corrigidos para o ano de 2025?

Resultados

I. Os resultados apresentados no material suplementar e nas tabelas do manuscrito estão muito bem detalhados, dispensando assim sugestões ou esclarecimentos adicionais.

II. No trecho “Em estudo sobre preços de aquisição pública de medicamentos do grupo 1B do Componente Especializado da Assistência Farmacêutica pelas secretarias estaduais de saúde, o Paraná esteve entre os estados que obtiveram, comparativamente, preços mais baixos de aquisição”, qual foi esse estudo? Não há citação bibliográfica no texto.

Discussão

I. Considerando que há existência de elenco complementar das secretarias estaduais de saúde e isso em geral, corresponde à extensão de uso para situações clínicas ou critérios não contemplados nos Protocolo Clínico e Diretrizes Terapêuticas do Sistema Único de Saúde, não seria conveniente discutir que os valores gastos pelo Paraná possam estar superestimados? Em uma visão reducionista, uma vez que os estados podem dispor de tais listas, é de se esperar que os mesmos tenham um gasto para além daquele que ocorreria caso fosse comprado em maiores unidades pelo Ministério da Saúde?

